# The relationship of postpartum depression with dyadic coping strategies and relationship satisfaction

**DOI:** 10.1590/1806-9282.20251893

**Published:** 2026-06-29

**Authors:** Hüsna Ekin Ateş, Seyhan Çankaya, Bahar Özdemir

**Affiliations:** 1 Selcuk University, Aladdin Keykubat Campus Selcuklu, Faculty of Health Science, Department of Midwifery – Konya, Turkey.; 2 KTO Karatay University, Faculty of Medicine, Department of Surgical Medical Sciences, Division of Obstetrics and Gynecology – Karatay, Konya.

**Keywords:** Parenting, Family relations, Depression, Postpartum, Coping inventory for stressful situations

## Abstract

**OBJECTIVE:**

The aim of this study was to determine the relationship between postpartum depression, dyadic coping strategies, and relationship satisfaction.

**METHODS:**

This study was designed as a descriptive cross-section. The study was conducted in 10 Family Health Centers in Southern Turkey. The study included 285 mothers who were registered to these Family Health Centers and who had babies aged 1–12 months. Interviews with mothers were held in a private room at the Family Health Centers between June 1, 2022, and June 1, 2023, and the data were collected in this room. Data were collected using a personal information form, the Dyadic Coping Inventory, the Relationship Assessment Scale, and the Edinburgh Postnatal Depression Scale.

**RESULTS:**

In the study, we found that 28.4% (n=81) of the mothers had postpartum depression. It was found that mothers with postpartum depression had significantly lower self-dyadic coping behaviors, and their partners had lower dyadic coping behaviors and lower joint dyadic coping behaviors than those of mothers without postpartum depression (p<0.05). The relationship satisfaction of mothers with postpartum depression was significantly lower than that of mothers without postpartum depression (p<0.05). As a result of the regression analysis, the mother's self-dyadic coping behaviors, partner's dyadic coping with stress, and relationship satisfaction were found to be significant risk factors for postpartum depression (p<0.01).

**CONCLUSION:**

In conclusion, mothers with poor dyadic coping strategies and low relationship satisfaction were more likely to experience postpartum depression.

## INTRODUCTION

The postpartum period is a time of significant physical, social, and emotional change for mothers and their families. During this transition, challenges such as adjusting to motherhood, baby care, fatigue, hormonal fluctuations, and disrupted routines can negatively affect maternal psychological adjustment^1^. Increased stress during this period has been linked to higher depressive symptoms^2^.

According to the Diagnostic and Statistical Manual of Mental Disorders, Fifth Edition (DSM-V), postpartum depression (PPD) is classified as a non-psychotic major depressive episode occurring during pregnancy or within one month after birth^3^. PPD is a common perinatal psychiatric disorder with serious consequences for both mother and infant, characterized by depressed mood, loss of interest, sleep and appetite disturbances, feelings of guilt, and suicidal ideation^4,5^. Its prevalence is approximately 24% in Turkey and 17% worldwide^6,7^.

The postpartum period also poses relational challenges for couples, including fatigue, parenting decisions, and changing roles. Dyadic coping refers to the processes through which partners provide mutual support and collaboratively manage stressors encountered within the relationship. This construct plays a significant role in promoting emotional adjustment, enhancing parenting self-efficacy, and fostering relationship satisfaction, as it reflects the couple's ability to function as a unified system in coping with individual and shared challenges^8^. Studies show that positive dyadic coping reduces depressive symptoms and enhances marital harmony, whereas poor communication and low partner support increase PPD symptoms^9,10^.

In Turkey's predominantly patriarchal family structure, limited communication and traditional gender roles may hinder effective stress coping and relationship satisfaction^11^. Therefore, examining PPD within the context of dyadic coping and relationship satisfaction offers a broader understanding of postpartum mental health.

This study aims to investigate the effects of mothers’ dyadic coping with stress and relationship satisfaction on postpartum depression, emphasizing the couple's mutual and interactive role in maternal psychological adjustment.

## METHODS

### Study design

This study was designed as a descriptive study.

### Setting

The study was conducted in 10 Family Health Centers (FHCs) located in the central districts of Antalya, southern Turkey. Antalya has five central districts with a total of 143 FHCs: Kepez (53), Muratpaşa (50), Konyaaltı (19), Döşemealtı (10), and Aksu (11). To ensure socioeconomic diversity, two FHCs representing moderate and low socioeconomic levels were selected from each district, and data were collected from mothers registered in these centers.

### Participants

Mothers registered at the selected FHCs who had healthy infants aged 1–12 months and met the study criteria were included. Inclusion criteria were being 18 years or older, married or living with a partner, having a singleton and healthy infant aged 1–12 months, being able to read and understand Turkish, and volunteering to participate. Mothers with a history of depression or other mental disorders, high-risk pregnancy, fetal anomaly, alcohol or substance abuse, perinatal loss or stillbirth, postpartum complications (e.g., hemorrhage, infection, mastitis, thromboembolic disease), or intellectual/visual/hearing impairment were excluded.

### Study population

The population of the study consisted of mothers who were registered to 10 FHCs in the city center of Antalya between June 01, 2022, and June 01, 2023, and who had babies aged 1–12 months.

### Sampling and methodology of the study

The sample size of the study was calculated based on the prevalence of PPD (26.5%) reported in Çankaya and Ataş, using the G-power 3.1.9.2 program, considering a one-unit difference, 95% power, 0.05 margin of error, and 0.1 effect size. It was calculated that 282 mothers should be included^12^. Mothers were included in the study according to the disproportionate stratified sampling method.

### Data collection method

Before data collection, mothers were informed about the study's purpose and procedures. The interviews were conducted in private rooms within FHCs, and the data were obtained from the women through self-report by the researchers. Participants were assured of confidentiality, anonymity, and voluntary participation, and no incentives were provided. Completing the forms took approximately 10–15 min. A pilot test was conducted with 30 mothers (10%) to assess the clarity of the data collection forms, and these participants were excluded from the main sample.

### Data collection tools

A Personal Information Form developed by the researcher in line with the literature, the Dyadic Coping Inventory (DCI), the Relationship Assessment Scale (RAS), and the Edinburgh Postnatal Depression Scale (EPDS) were used to collect the data.

#### Personal information form

The Personal Information Form, developed by the researcher based on the literature, consists of two sections. The first includes eight items on mothers’ sociodemographic characteristics (e.g., age, education, income perception, family type, and duration of marriage). The second comprises 14 items on obstetric characteristics, including the number of pregnancies, births, living children, planned pregnancy status, baby's gender, parental satisfaction with the baby's gender, difficulty in infant care, and adaptation to the maternal role^11,12^.

#### Dyadic coping inventory

The DCI was developed to assess stress communication and dyadic coping in romantic relationships^13^. It is a 37-item, five-point Likert-type scale (1=never, 5=always) with 12 subdimensions covering self-perception, partner perception, and joint coping. The inventory yields three total scores reflecting self, partner, and joint coping, and the subscales can be evaluated separately. The Turkish adaptation and reliability study was conducted by Kurt and Akbaş, reporting Cronbach's alpha values of 0.68 for self, 0.78 for partner, and 0.84 for joint coping^14^. In the present study, internal consistency coefficients were 0.76, 0.87, and 0.88, respectively, with an overall Cronbach's alpha of 0.92.

#### Relationship Assessment Scale

The RAS was developed to evaluate individuals’ satisfaction in romantic relationships, with an internal consistency coefficient of 0.86^15^. The seven-item, seven-point Likert-type scale (1–7) includes two reverse-scored items (4 and 7), and total scores range from 7 to 49, with higher scores indicating greater satisfaction. The Turkish adaptation demonstrated a Cronbach's alpha of 0.86^16^. In the present study, the internal consistency coefficient was 0.77.

#### Edinburgh Postnatal Depression Scale

The EPDS is a self-report tool developed to assess the risk and severity of postpartum depression^17^. It consists of 10 Likert-type items, with a total score ranging from 0 to 30; higher scores indicate greater depression risk. Items 3, 5–10 are reverse scored (3–0), while items 1, 2, and 4 are scored 0–3. A cut-off score of 13 or higher indicates risk for postpartum depression. The Turkish validity and reliability study reported a Cronbach's alpha of 0.79^18^, while in the present study, it was 0.86.

### Data analysis

Data analysis was performed using Number Cruncher Statistical System (NCSS) 2020 Statistical Software (NCSS LLC, Kaysville, Utah, USA) with support from a statistician. Quantitative variables were summarized as mean, standard deviation, median, minimum, and maximum, while qualitative variables were expressed as frequency and percentage. Normality was assessed using the Shapiro-Wilk test and Box Plot graphs. Student's t-test was used for comparisons between two normally distributed groups, and Binary Logistic Regression Analysis identified risk factors associated with the EPDS cut-off score. Results were evaluated at a 95%CI and a significance level of p<0.05.

### Ethical considerations

Ethical approval for the study was obtained from the local ethics committee (Registration number: #2022/55) and the Antalya Provincial Health Directorate (Institution approval number: #05.04.2022-266373). Permissions for the use of all study scales were secured. Eligible mothers were fully informed about the study and assured that participation was voluntary and withdrawal could occur at any time without consequence. The study was conducted in accordance with the ethical principles of the 1964 Declaration of Helsinki and its later amendments. All study expenses were covered by the researcher without external funding.

## RESULTS

The sociodemographic and obstetric characteristics of the mothers are presented in Table 1. Participants’ ages ranged from 19 to 46 years (mean 30.0±4.8), and all were married, with an average marriage duration of 5.5±4.4 years. All mothers had term, healthy infants, who were on average 5.8±3.6 months old. About 20.7% (n=59) reported difficulties with infant care, and 62.8% (n=179) received partner support in breastfeeding, childcare, and household tasks. Overall, 51.2% (n=146) adapted well to the maternal role. No significant associations were found between PPD and maternal sociodemographic or obstetric characteristics.

**Table 1:** Distribution of sociodemographic and obstetric characteristics of mothers (n=285).

		Mean (SD)	Median (min–max)
Age		30 (4.8)	29 (19–46)
Year of marriage		5.5 (4.4)	4 (1–29)
Number of pregnancies		1.8 (1.1)	1 (1–7)
Number of births		1.6 (0.9)	1 (1–6)
Number of living children		1.6 (0.9)	1 (1–6)
Time elapsed after delivery (months)		5.8 (3.6)	6 (1–12)
		n	%
Education status			
	Primary school	22	7.7
	High school	83	29.1
	University and above	180	63.2
Employment status			
	Working	280	98.2
	Not working	5	1.8
Family type			
	Nuclear family	258	90.5
	Extended family	27	9.5
Mother's perception of monthly income			
	Good	81	28.4
	Moderate	192	67.4
	Bad	12	4.2
Attending regular follow-up visits during pregnancy			
	Yes	257	90.2
	No	28	9.8
Wanted pregnancy			
	Yes	255	89.5
	No	30	10.5

SD: standard deviation.

The mean DCI, RAS, and EPDS scores of the mothers are presented in Table 2. The mean self DCI score was 58.5±7.7, partner DCI 55.8±10.3, and joint DCI 26.0±6.2, with a total DCI score ranging from 72 to 184 (mean 140.3±21.1). The mean RAS score was 37.2±7.4. EPDS scores ranged from 0 to 26, with a mean of 9.9±5.9, and 28.4% (n=81) of mothers were identified as having postpartum depression.

**Table 2:** Comparison of mothers’ postpartum depression status with Dyadic Stress Coping Inventory total score and sub-dimensions and Relationship Assessment Scale scores.

		Without PPD (DSCI≤12)	With PPD (EPDS≥13)	p-value
Total DSCI	Mean (SD)	146.2±17.9	125.3±21.3	**<0.001**
	Median (min–max)	147 (82–184)	126 (72–171)	
Self DSCI	Mean (SD)	60.1±7	54.3±7.8	**<0.001**
	Median (min–max)	60 (43–75)	56 (23–74)	
Partner's DSCI	Mean (SD)	58.5±8.4	49±11.5	**<0.001**
	Median (min–max)	59 (26–75)	49 (27–71)	
Joint DSCI	Mean (SD)	27.6±5.2	22±6.9	**<0.001**
	Median (min–max)	28 (13–35)	23 (7–35)	
Total RAS	Mean (SD)	39.1±5.8	32.5±8.7	**<0.001**
	Median (min–max)	40 (16–49)	35 (13–48)	

Student's t-test. Bold value: p<0.05 is a statistically significant value. DSCI, Dyadic Stress Coping Inventory; Self DSCI, Self Dyadic Stress Coping Inventory; Partner's DSCI, Partner's Dyadic Stress Coping Inventory; Joint DSCI, Joint Dyadic Stress Coping Inventory; RAS, Relationship Assessment Scale; EPDS, Edinburgh Postnatal Depression Scale; PPD, postpartum depression; SD: standard deviation.

Table 2 presents the comparison of mothers’ PPD status with DCI total and subdimension scores and RAS scores. Mothers with PPD had significantly lower stress coping scores (self, partner, and joint DCI) than those without PPD (p<0.01), and their couple relationship satisfaction was also significantly lower (p<0.001).

Backward Binary Logistic Regression analysis of factors affecting PPD is presented in Table 3. The model examining self DCI, partner DCI, joint DCI, and total RAS scores was significant (χ^2^=67.097, p<0.001), explaining 30% of the variance (Nagelkerke R^2^=0.301) with a correct classification rate of 76.8%. In the model, self DCI (OR 0.952, 95%CI 0.906–0.999), partner DCI (OR 0.948, 95%CI 0.914–0.984), and total RAS (OR 0.929, 95%CI 0.886–0.973) were significant predictors of PPD (p<0.05). A one-unit increase in PPD risk corresponded to decreases of 0.050 in self DCI, 0.053 in partner DCI, and 0.074 in couple relationship satisfaction (p<0.05).

**Table 3:** Results of backward binary logistic regression analysis of risk factors affecting postpartum depression.

		B	SD	p	Odds ratio (OR)	95%Cl	
						Lower	Upper
Step 2^a^	Self DSCI score	-0.050	0.025	**0.046**	0.952	0.906	0.999
	Partner's DSCI score	-0.053	0.019	**0.005**	0.948	0.914	0.984
	Joint DSCI score	-0.030	0.039	0.438	0.970	0.899	1.047
	RAS score	-0.074	0.024	**0.002**	0.929	0.886	0.973

Bold value: p<0.05 is a statistically significant value. Performed with the backward method binary logistic regression analysis. Self DSCI, Self Dyadic Stress Coping Inventory; Partner's DSCI, Partner's Dyadic Stress Coping Inventory; Joint DSCI, Joint Dyadic Stress Coping Inventory; RAS, Relationship Assessment Scale; PPD, postpartum depression; SD: standard deviation; OR: odds ratio; CI: confidence interval.

a Model obtained at the second step of the backward elimination procedure.

## DISCUSSION

This study examined the effects of dyadic coping with stress and relationship satisfaction on PPD in mothers. The prevalence of PPD in this sample was 28.4%, consistent with prior studies in Turkey reporting rates between 3.5 and 58%^6^. Given the high prevalence, early identification of risk factors and psychological support for mothers is crucial^19^.

The postpartum period represents a "dyadic stress context," affecting both partners^20^. In line with previous research, mothers with PPD exhibited significantly lower self, partner, and joint dyadic coping scores compared to mothers without PPD^21,22^. Partner support and active engagement in dyadic coping were identified as protective factors against PPD, highlighting the importance of emotion- and problem-focused coping strategies during this period^23^. Joint coping behaviors, including shared problem-solving, emotional sharing, and mutual commitment, were also lower in mothers with PPD, consistent with evidence linking joint dyadic coping to better individual and parental adjustment^8,23^.

Relationship satisfaction was another significant predictor of PPD. Mothers with lower satisfaction reported higher depressive symptoms, reflecting findings that poor couple adjustment, increased conflict, and reduced positive interactions contribute to maternal PPD^23,24^. Interventions targeting couple-based stress management and relationship enhancement have been shown to improve maternal mental health and reduce negativity in couples^3,25^. Overall, this study highlights that coping with stress is a shared process, and both dyadic coping strategies and relationship satisfaction play crucial roles in protecting maternal psychological well-being during the postpartum period. While results are limited to one province and may not be nationally generalizable, the inclusion of diverse participants across 10 FHCs supports the applicability of findings within the region.

## CONCLUSION

In this study, 28.4% of mothers were identified with PPD. Lower self-dyadic coping, poorer perception of partner's dyadic coping, and reduced relationship satisfaction were significant risk factors. Mothers with PPD had significantly lower self, partner, and joint dyadic coping scores compared to those without PPD.

## Data Availability

The datasets generated and/or analyzed during the current study are available from the corresponding author upon reasonable request.

## References

[1] Abhari ZH, Karimi FZ, Mazloom SR, Taghizdeh Z, Asghari Nekah SM (2021). Effect of counseling based on Gamble's approach on postpartum anxiety in primiparous women. J Midwifery Reprod Health.

[2] Ghaedrahmati M, Kazemi A, Kheirabadi G, Ebrahimi A, Bahrami M (2017). Postpartum depression risk factors: a narrative review. J Educ Health Promot.

[3] American Psychiatric Association (2016). Diagnostic and statistical manual of mental disorders.

[4] Hahn-Holbrook J, Cornwell-Hinrichs T, Anaya I (2018). Economic and health predictors of national postpartum depression prevalence: a systematic review, meta-analysis, and meta-regression of 291 studies from 56 countries. Front Psychiatry.

[5] Mughal S, Azhar Y, Siddiqui W, May K (2022). StatPearls [Internet].

[6] Karaçam Z, Çoban A, Akbaş B, Karabulut E (2018). Status of postpartum depression in Turkey: a meta-analysis. Health Care Women Int.

[7] Shorey S, Chee CYI, Ng ED, Chan YH, Tam WWS, Chong YS (2018). Prevalence and incidence of postpartum depression among healthy mothers: a systematic review and meta-analysis. J Psychiatr Res.

[8] Falconier MK, Jackson JB, Hilpert P, Bodenmann G (2015). Dyadic coping and relationship satisfaction: a meta-analysis. Clin Psychol Rev.

[9] Rottmann N, Hansen DG, Larsen PV, Nicolaisen A, Flyger H, Johansen C (2015). Dyadic coping within couples dealing with breast cancer: a longitudinal, population-based study. Health Psychol.

[10] Köse Tuncer S, Kaloğlu Binici D (2022). The correlation between spousal support and postpartum depression in fathers. Perspect Psychiatr Care.

[11] Ünal Ö, Akgün S (2022). Relationship of conflict resolution styles in marriage with marital adjustment and satisfaction. Curr Approach Psychiatry.

[12] Çankaya S, Ataş A (2022). Factors affecting postpartum depression in Turkish women. Arch Psychiatr Nurs.

[13] Bodenmann G (2008). Dyadisches Coping Inventar (DCI). Test manual [Dyadic Coping Inventory (DCI). Test manual].

[14] Kurt İE, Akbaş T (2019). Adaptation of dyadic coping inventory into Turkish. OPUS Int J Soc Res.

[15] Hendrick SS, Dicke A, Hendrick C (1998). The Relationship Assessment Scale. J Soc Pers Relationships.

[16] Curun F (2001). The effects of sexism and sex role orientation on romantic relationship satisfaction.

[17] Cox JL, Holden JM, Sagovsky R (1987). Detection of postnatal depression. Development of the 10-item Edinburgh Postnatal Depression Scale. Br J Psychiatry.

[18] Engindeniz N, Küey L, Kültür S (1996). Spring Symposium.

[19] World Health Organization (2015). Maternal mental health.

[20] Bodenmann G, Falconier M, Randall AK, Lebow J, Chambers A, Breunlin D (2017). Encyclopedia of couple and family therapy.

[21] Çankaya S, Buran G (2023). The effects of dyadic coping strategies and dyadic conflict resolution styles on postpartum depression of mothers in heterosexual marriages in Turkey. Bull Menninger Clin.

[22] Stadelmann C, Senn M, Forster F, Rauch-Anderegg V, Nussbeck FW, Johnson MD (2023). Dyadic coping trajectories across the transition to parenthood: associations with child mental health problems. J Fam Psychol.

[23] Alves S, Fonseca A, Canavarro MC, Pereira M (2020). Does dyadic coping predict couples’ postpartum psychosocial adjustment? A dyadic longitudinal study. Front Psychol.

[24] Ngai FW, Lam W (2021). Stress, marital relationship and quality of life of couples across the perinatal period. Matern Child Health J.

[25] Barbato A, D’Avanzo B, Parabiaghi A (2018). Couple therapy for depression. Cochrane Database Syst Rev.

